# Improving chronic kidney disease detection and treatment in the United States: the chronic kidney disease cascade of care (C^3^) study protocol

**DOI:** 10.1186/s12882-022-02943-z

**Published:** 2022-10-12

**Authors:** Julio A. Lamprea-Montealegre, Priya Joshi, Abigail S. Shapiro, Erin Madden, Krista Navarra, O. Alison Potok, L. Parker Gregg, Tanya Podchiyska, Amy Robinson, Mary K. Goldstein, Carmen A. Peralta, Simerjot K. Jassal, Sankar D. Navaneethan, Dena. E. Rifkin, Virginia Wang, Michael G. Shlipak, Michelle M. Estrella

**Affiliations:** 1 University of California San Francisco, 4150 Clement St., Building 2, Room 145, San Francisco, CA 94121 USA; 2 Kidney Health Research Collaborative, Department of Medicine, University of California, San Francisco, CA USA; 3 San Francisco VA Health Care System, San Francisco, CA USA; 4 Duke University, Durham, NC USA; 5 Durham VA Health Care System, Durham, NC USA; 6 University of California San Diego, La Jolla, CA USA; 7 San Diego VA Health Care System, San Diego, CA USA; 8 Baylor College of Medicine, Michael E. DeBakey VA Medical Center, Houston, TX USA; 9 Veterans Affairs Health Services Research and Development Center for Innovations in Quality, Effectiveness, and Safety and Section of Nephrology, Michael E. DeBakey VA Medical Center, TX Houston, USA; 10 VA Palo Alto Health Care System, Palo Alto, CA USA; 11 VA Sierra Pacific Network (VISN 21), Pleasant Hill, CA USA; 12 Stanford University, Stanford, CA USA; 13 Cricket Health, Inc, San Francisco, CA USA

**Keywords:** Kidney diseases, Cardiovascular diseases, Disease management, Implementation science

## Abstract

**Background:**

There are major gaps in the implementation of guideline-concordant care for persons with chronic kidney disease (CKD). The CKD Cascade of Care (C^3^) initiative seeks to improve CKD care by improving detection and treatment of CKD in primary care.

**Methods:**

C^3^ is a multi-modal initiative deployed in three major academic medical centers within the Department of Veterans Affairs (VA) Health Care System: San Francisco VA, San Diego VA, and Houston VA. The main objective of the first phase of C^3^ described in this protocol is to establish the infrastructure for universal CKD detection among primary care patients at high-risk for CKD with a triple-marker screen comprising cystatin C, creatinine, and albuminuria. Across the three sites, a comprehensive educational intervention and the integration of primary care-based clinical champions will be employed with the goal of improving CKD detection and treatment. The San Francisco VA will also implement a practice-facilitation intervention leveraging telehealth and health informatics tools and capabilities for enhanced CKD detection. Parallel formative evaluation across the three sites will assess the feasibility and acceptability of integrating cystatin C as part of routine CKD detection in primary care practice. The effectiveness of the interventions will be assessed using a pre-post observational design for change in the proportion of patients tested annually for CKD. Secondary outcomes will assess change in the initiation of cardio-kidney protective therapies and in nephrology referrals of high-risk patients.

**Discussion:**

The first phase of C^3^ is a multi-facility multi-modal initiative that aims to improve CKD care by implementing a triple-marker screen for enhanced CKD detection in primary care.

## Background

Chronic kidney disease (CKD), defined as an estimated glomerular filtration rate (eGFR) less than 60 ml/min/1.73 m^2^ and/or a urinary albumin to creatinine ratio (ACR) greater than or equal to 30 mg/g, is a major public health problem. Globally, nearly 10% of the adult population has a diagnosis of CKD which is associated with substantially increased risks for cardiovascular events, progression to end-stage kidney disease (ESKD) requiring dialysis or transplant, in-hospital complications, and early death [1]. The healthcare costs of CKD are substantial. In 2018, overall Medicare fee for service spending for patients with CKD exceeded $81 billion, representing 23% of total Medicare spending [2].

Most preventive guidelines recommend testing for CKD as part of the usual management of high-risk conditions including diabetes, hypertension and established cardiovascular disease [3, 4]. The rationale for these recommendations is that detection and staging of CKD are critical for proper kidney and cardiovascular risk stratification, prompt referral to nephrology care, and initiation of cardio-kidney preventive therapies. Collectively, these actions could vastly lower the adverse health consequences of CKD, but major evidence-to-care gaps exist in CKD detection which is the necessary first step towards optimizing clinical management and outcomes. For instance, less than 50% of patients with diabetes undergo regular testing for CKD with eGFR and albuminuria [5]. For patients with hypertension but without diabetes, testing for albuminuria is routinely conducted in less than 10% of patients [6]. As a result, CKD at early stages is poorly recognized, marking a missed opportunity to treat CKD in patients when preventive efforts could have the largest impact [7].

Prior research has identified multi-level barriers for the optimal detection and treatment of CKD in primary care. Primary care providers (PCPs) have reported limited knowledge of CKD and its complications and low awareness of guidelines for CKD care [8]. In addition, PCPs have reported low self-efficacy in educating and treating patients with CKD [8]. From a system-level perspective, PCPs have reported limited time and clinical support to care for patients with CKD who often have competing medical priorities [8, 9]. There is also substantial heterogeneity in the assessment and reporting of albuminuria and limited availability of cystatin C as a marker of kidney function to enhance CKD detection and staging. Lack of institutional support for CKD care improvement initiatives may further widen this care gap. While our research group has demonstrated that comprehensive CKD detection and treatment is feasible in primary care [10], larger implementation efforts are needed to bridge large evidence to care gaps.

The CKD Cascade of Care (C^3^) is a multi-modal intervention developed and implemented within the Department of Veterans Affairs (VA) Health Administration aimed at improving CKD detection, risk-stratification, and treatment in primary care. The first phase of the C^3^ initiative which is described in this protocol seeks to improve the appropriate use of universal testing for CKD among patients with type 2 diabetes (T2D), hypertension or cardiovascular disease in primary care through the establishment of a triple-marker screen for CKD: creatinine-based eGFR, cystatin C-based eGFR, and albuminuria assessed by the ACR. The inclusion of cystatin C in the routine assessment of CKD follows the VA/Department of Defense (VA/DoD) Guidelines and the Kidney Disease Global Outcomes (KDIGO) Controversies Conference recommendations for CKD early detection, risk stratification and treatment [11, 12]. Compared to GFR estimated by creatinine, cystatin C-based eGFR is more strongly associated with CVD events [13, 14]. Thus, its inclusion in the routine assessment of CKD may enhance CVD risk-stratification. In addition, GFR estimated with cystatin C does not include a race coefficient, which has sparked controversy for creatinine-based equations since race is a social and not a biological construct.

Because of the novelty in introducing cystatin C for the detection and staging of CKD in clinical practice, this project establishes the infrastructure to facilitate and promote appropriate utilization of cystatin C testing to facilitate improvements in disease recognition and clinical management. We also assess the acceptability and feasibility of cystatin C use in primary care practice to inform potential benefits and challenges of expanded implementation of cystatin C testing in the VA.

## Methods

### Study setting and context

C^3^ was developed in response to VA Directive 1053 on CKD prevention, early recognition and treatment, issued in March 2020, which recommended improvements to CKD care programs nationwide [15]. Suggested practices include: (1) implementing CKD programs within existing primary care capabilities; (2) fostering a collaborative environment between primary care and specialty providers to improve CKD care; and (3) implementing comprehensive screening programs for CKD detection consistent with the VA/DoD clinical practice guidelines. Importantly, the guidelines also recommended the addition of cystatin C to creatinine and albuminuria testing for the initial diagnosis and staging of CKD [11]. As one of the only medical centers with existing cystatin C testing capabilities, the San Francisco VA was at the vanguard of implementing these system-level goals and led the development of the C^3^ initiative, working with other VA hospitals and their local laboratory directors to establish on-site cystatin C testing for the San Diego and Houston VAs. In the San Diego VA, local cystatin C testing commenced on January 2021 and in the Houston VA on July 2021. The Institutional Review Board of the University of California San Francisco approved the waiver of informed consent.

In line with the national VA recommendations, the goal of C^3^ is to implement a triple-marker screen for CKD detection in patients at high-risk for CKD in primary care. This will be conducted in the primary care clinics of three VA medical centers: San Francisco VA, San Diego VA, and Houston VA which account for a large multi-ethnic population of patients with a substantial CKD burden and high rate of concurrent comorbidities (Table 1). In the VA system, primary care is structured in patient-centered medical homes called Patient Aligned Care Teams (PACTs) each consisting of a physician, a nurse practitioner, and a medical assistant. Across the three medical centers, there are 115 PCPs, which include physicians, nurse practitioners and physician assistants with prescription privileges: 25 PCPs in the San Francisco VA, 34 PCPs in the San Diego VA, and 56 PCPs in the Houston VA**.** Supporting each PACT, the clinics at all sites have embedded services including clinical pharmacists, case managers, and social workers. All three sites are major teaching facilities with participating internal medicine and nurse practitioner residents and medical students.

**Table 1:** Characteristics of patients attending primary care in the San Francisco, San Diego, and Houston VAs in 2020

Characteristic	**San Francisco VA** **(*n* = 21,701)**	**San Diego VA** **(*n* = 51,113)**	**Houston VA** **(*n* = 79,526)**
**Age; mean** (SD)	65 (16)	56 (17)	59 (16)
**Female;** n (%)	1,720 (8)	6,475 (13)	9,959 (12)
**Race/Ethnicity**; n (%)			
White	13,760 (63)	30,178 (59)	47,409 (60)
Black	1,874 (9)	8,027 (16)	26,724 (34)
Asian	1,298 (6)	5,822 (11)	772 (1)
Alaska Native/American Indian	235 (1)	521 (1)	340 (0.4)
Native Hawaiian or other Pacific Islander	296 (1)	1179 (2)	727 (1)
Hispanic	1,270 (6)	8,629 (17)	8,955 (11)
**Co-morbidities**			
Type 2 diabetes; n (%)	4,255 (20)	9,902 (19)	22,437 (28)
Hypertension; n (%)	18,837 (87)	39,552 (77)	69,255 (87)
Cardiovascular disease; n (%)	4,759 (22)	6,722 (13)	16,240 (20)
Chronic Kidney Disease; n (%)	3,701 (17)	6,143 (12)	10,339 (13)
**CKD testing, by co-morbidity subgroups**			
**Type 2 diabetes**	***N* = 4,255**	***N* = 9,902**	***N* = 22,437**
eGFR creatinine; n (%)	3,613 (85)	8,718 (88)	19,601 (87)
eGFR cystatin C; n (%)	194 (5)	NA	NA
ACR; n (%)	2,389 (56)	5,327 (54)	665 (3)
PCR; n (%)	135 (2)	533 (5)	662 (3)
Urine albumin without creatinine	34 (0.8)	1,001 (10)	11,044 (49)
Urine protein without creatinine	1,541 (36)	48 (0.5)	14,504 (64)
**Hypertension without type 2 diabetes**	***N* = 14,697**	***N* = 30,057**	***N* = 47,462**
eGFR creatinine; n (%)	9,877 (67.2)	22,513 (74.9)	3,6878(77.7)
eGFR cystatin C; n (%)	306 (2)	NA	NA
ACR; n (%)	1,595 (10)	4,465 (14.8)	329 (0.7)
PCR; n (%)	191 (1.3)	426 (1.4)	575 (1.2)
Urine albumin without creatinine; n (%)	32 (0.2)	2,111 (7.0)	12,150 (25.6)
Urine protein without creatinine; n (%)	4,413 (30.0)	83 (0.3)	27,132 (57.2)
**Cardiovascular disease without type 2 diabetes**	***N* = 3,254**	***N* = 4,028**	***N* = 8,578**
eGFR creatinine; n (%)	2,405 (73.9)	,3376 (83.8)	7,180 (83.7)
eGFR cystatin C; n (%)	112 (3.4)	NA	NA
ACR; n (%)	430 (13.2)	687 (17.0)	94 (1.1)
PCR; n (%)	80 (2.5)	153 (3.8)	182 (2.1)
Urine albumin without creatinine; n (%)	4 (0.1)	202 (5.0)	2,253 (26.3)
Urine protein without creatinine; n (%)	1,104 (33.9)	16 (0.4)	5,188 (60.5)
**CKD treatment**			
Patients with albuminuria on ACEI/ARB; n (%)	1,339 (78.1%)	2,448 (75.2)	1,150 (86.7)
Patients with albuminuria on SGLT2i; n (%)	130 (7.6)	431 (13.2)	93 (7.0)

*CKD* Chronic kidney disease, *eGFR* Estimated glomerular filtration rate, *ACR* Urinary albumin to creatinine ratio, *PCR* Urinary protein to creatinine ratio, *ACEI/ARB* Angiotensin-converting enzyme inhibitors or angiotensin receptor blockers, *SGLT2i* Sodium-glucose co-transporter 2 inhibitors

*NA* Not applicable; cystatin C testing commenced at these sites in 2021

The primary outcome of the proposed interventions will be the improvement in the testing for CKD using a triple-marker screen comprising creatinine eGFR, cystatin C eGFR, and ACR among patients with T2D, hypertension, and cardiovascular disease. Because some of the interventions will provide specific guidance on appropriate nephrology referrals and on initiation of cardio-kidney protective therapies, secondary exploratory outcomes will assess the association between the intervention and initiation of cardio-kidney protective therapies and in the rate of nephology referrals over time.

### Pre-implementation activities

The C^3^ study team comprises a multi-disciplinary team of practitioners, clinical researchers, biostatisticians, and health services researchers/implementation scientists from across the three sites and from the Durham VA. The team has met monthly for the year prior to implementation and will continue to meet monthly during the study period. The team has provided input on the content of the educational strategy, the recruitment and training of the primary care clinic champions, and the practice facilitation intervention at the San Francisco VA. The team has also provided input on the design of the questionnaire for the formative evaluations on cystatin C testing.

### Infrastructure for cystatin C testing in San Diego and Houston VAs

For each site, the local laboratory lead and assistant personnel, along with Beckman Coulter®, developed the infrastructure for on-site cystatin C testing capabilities. At the San Diego VA, these activities involved connecting with the lead physician in laboratory medicine (Dr. Jessica Wang-Rodriguez) to develop an internal use scenario for cystatin C (previously available as a send-out test). Local excess serum samples for testing the assay at extreme values were provided from the hemodialysis population. Once the assay was internally validated, the lab order was added to the main laboratory menu. At the Houston VA, data generated from 10 random samples were validated by measuring cystatin C in the San Francisco VA for the same samples. Cystatin C became available for ordering by providers in January 2021 in the San Diego VA and in July 2021 in the Houston VA.

### Kidney health research collaborative data repository

The Kidney Health Research Collaborative (KHRC) (co-directed by the C^3^ Principal Investigators at the San Francisco VA) created a unified clinical data repository that integrates the VA electronic medical record data sources to enhance research initiatives aimed at improving the care of Veterans with or at high-risk for CKD. It has curated data from over 10 million Veterans since 1997. The repository is updated daily and uses validated algorithms for the ascertainment of medical conditions through electronic medical records. Using the repository, we characterized the demographic and clinical characteristics of patients receiving primary care and established the current state of CKD testing at the three sites (Table 1).

To characterize the CKD detection gap across the three sites, baseline assessment of CKD testing yielded low rates of cystatin C testing in the San Francisco VA, low albuminuria testing across the three sites, and substantial variability in albuminuria testing and reporting (Table 1). Specifically, although cystatin C has been available at the San Francisco VA since 2012, less than 5% of patients were ordered to undergo cystatin C testing for CKD detection in 2020. In addition, although clinical guidelines recommend the ACR as the ideal test to assess albuminuria and clinical trials of cardio-kidney protective medications have used the ACR to guide treatment decisions, we found that less than 60% of patients with T2D were tested for ACR and that a significant proportion of tests are ordered as urine protein-to-creatinine ratio or urine albumin without urine creatinine. ACR testing for patients with hypertension or CVD without T2D is less than 20% across the three sites.

### Planned Implementation activities

#### Evaluation of initial experience of cystatin C testing in VA primary care

**Fig. 1:**
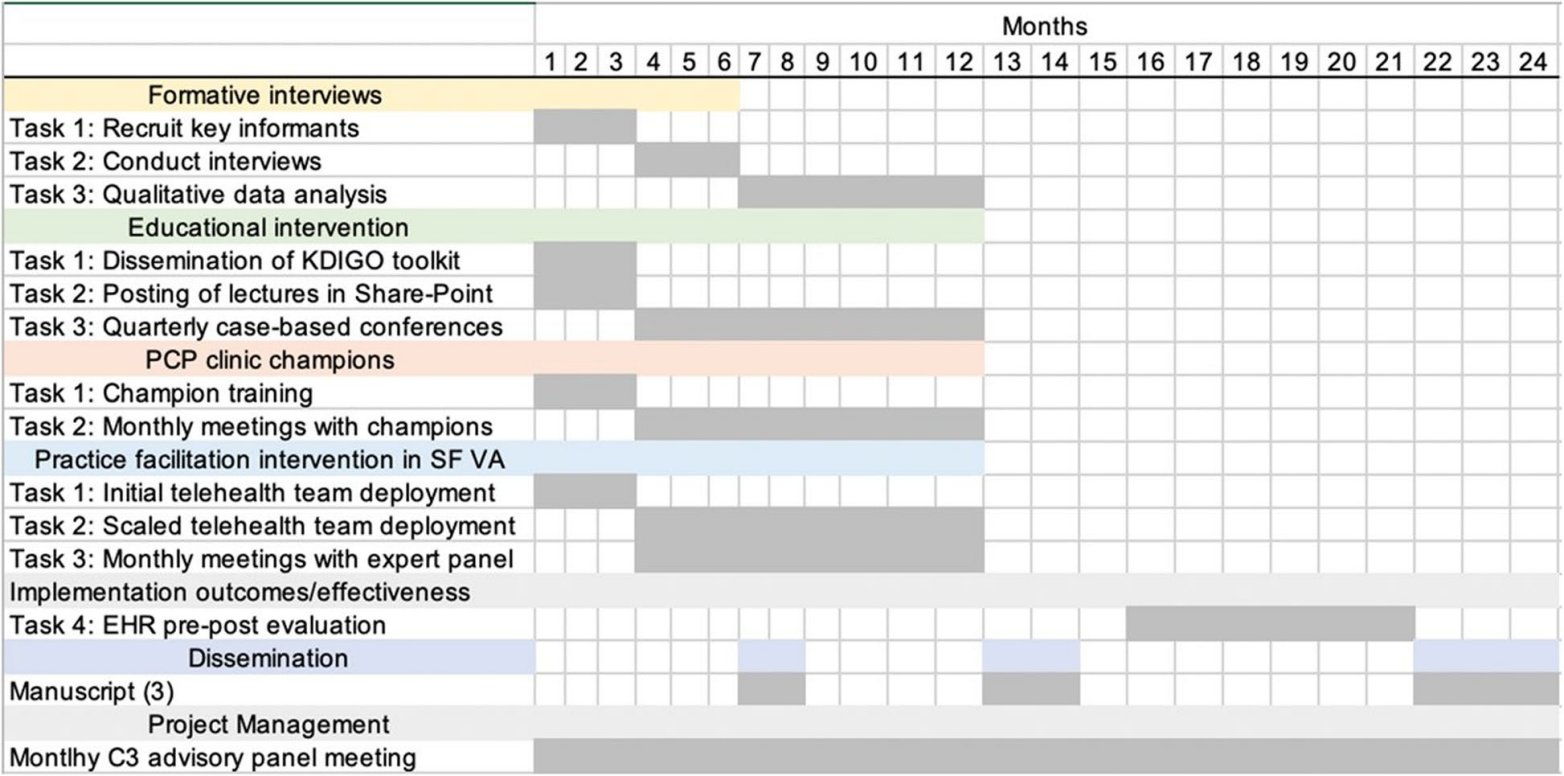
Study timeline

Qualitative explorations will assess the feasibility and acceptability of cystatin C deployment across the three sites and will run in parallel with the educational and practice-facilitation intervention. We will conduct a formative evaluation of implementation experience, through semi-structured interviews with PCPs, nephrologists, lab personnel, and other clinical support staff at each site (*n* = 12 per site, *N* = 36 across 3 sites). Information from these interviews will provide rich contextual data regarding patient-, provider- and system-level barriers to kidney disease testing and staging, particularly for cystatin C and urine albumin testing. Interviews will identify and examine factors important for effective intervention and their implementation [16]. These include provider perceptions regarding acceptability of the intervention, feasibility, and salience of intervention to their scope of work or clinical practice, as well as usability of the intervention content, format, and processes. The study timeline is shown in Fig. 1.

Participants will be recruited at their local sites via flyers, provider trainings, and faculty meetings and through an administrative data pull based on clinician location and role. A snowball sampling approach will then be used to identify additional key informants involved in kidney disease testing in VA primary care settings [17]. The semi-structured interview guide and interview protocols will be developed, pilot-tested, and approved by the C^3^ study team to ensure that questions draw responses to critical data from each respondent. Interviews will last 20–30 min and will be recorded and transcribed.

Transcripts will be reviewed, edited for accuracy, and summarized by two qualitative researchers, using Hamilton's rapid analysis approach to structure the qualitative inquiry [18]. The qualitative team will create a one-page summary template for the interviews using domains drawn from the interview guides and space for salient emerging ideas, de-identified participant information, and details and observations about the data collection episode. The qualitative analysts will summarize each transcript and any additional notes into this template and conduct a reflexive assessment of intra- and inter-coder reliability of randomly selected template segments compared with transcripts and notes. The qualitative team’s positionality as non-clinicians provides an outsider perspective on primary care; regular meetings with the project’s clinician investigators will occur to reduce bias and ensure data validity in the clinical context.

Summaries will be analyzed using matrix analysis [19]. At least two researchers will be involved in the matrix analysis process and development of findings. The matrices will include domains derived from the interview guide and summary templates. The matrix framework will be developed and finalized through a process of discussion and consensus. Matrices will be iteratively compared with templated notes to ensure completeness. Findings will be reviewed by the qualitative team along with the clinician investigators to identify key and actionable findings most relevant to primary care practice to facilitate potential modifications to the C^3^ program for further deployment.

#### Comprehensive educational intervention

To support uptake of triple marker testing, the study team will develop and disseminate educational materials, educational conferences, and collective case-based educational sessions to all participating sites. These materials include:

- KDIGO educational booklet for CKD early identification and intervention: This booklet is a reference guide for providers on screening for CKD [20]. It is based on the KDIGO Conference on early identification and intervention recommendations. The booklet describes who should be screened for CKD and who should not, and details recommended tests and evidence-based treatments to manage CKD.
- Educational sessions: A total of four 30-min video recorded lectures will be posted at each site’s SharePoint which is widely used by VA physicians. The lectures will cover four specific topics: (1) CKD detection and staging; (2) use of cystatin C for CKD detection and risk stratification; (3) albuminuria for CKD detection and staging; and (4) CKD treatment algorithms to prevent CKD progression and cardiovascular disease.
- Collective case-based conferences: Quarterly conferences at each site will be facilitated by the PCP champions and will be case-based using the EFECT framework [21]: Elicit patient-centered narrative, Facilitate a reflective team discussion, Evaluate the clinical evidence, Create a shared care plan, and Track outcomes. Each conference will last 60 min and will present two patients in which CKD was newly detected and treatment was commenced based on the CKD diagnosis. Before the conference, each PACT team will suggest the patients to present. The C^3^ advisory panel and clinical champions will ultimately select the patients to be presented, maximizing the involvement of different specialty providers and clinical pharmacists, and the anticipated educational impact.

#### Primary care clinical champions

At each site, one PCP will be identified and trained to serve as a local CKD Clinic Champion or reference provider for CKD detection and treatment. Three training sessions will cover the KDIGO guidelines for CKD early detection and treatment. Each session will last 30 min and will be conducted remotely by a member of the C^3^ advisory panel. The champions will be empowered to promote CKD detection and treatment and to address PACT team questions on CKD detection and treatment. The clinic champion training sessions will be completed during the first 2 months of the intervention across the three sites. In the subsequent 10 months, monthly meetings with champions will identify themes that were not well covered in the initial training sessions (Fig. 1). The training for champions will include the KDIGO recommendations for appropriate nephrology referrals.

#### Additional practice facilitation intervention at the San Francisco VA

**Fig. 2:**
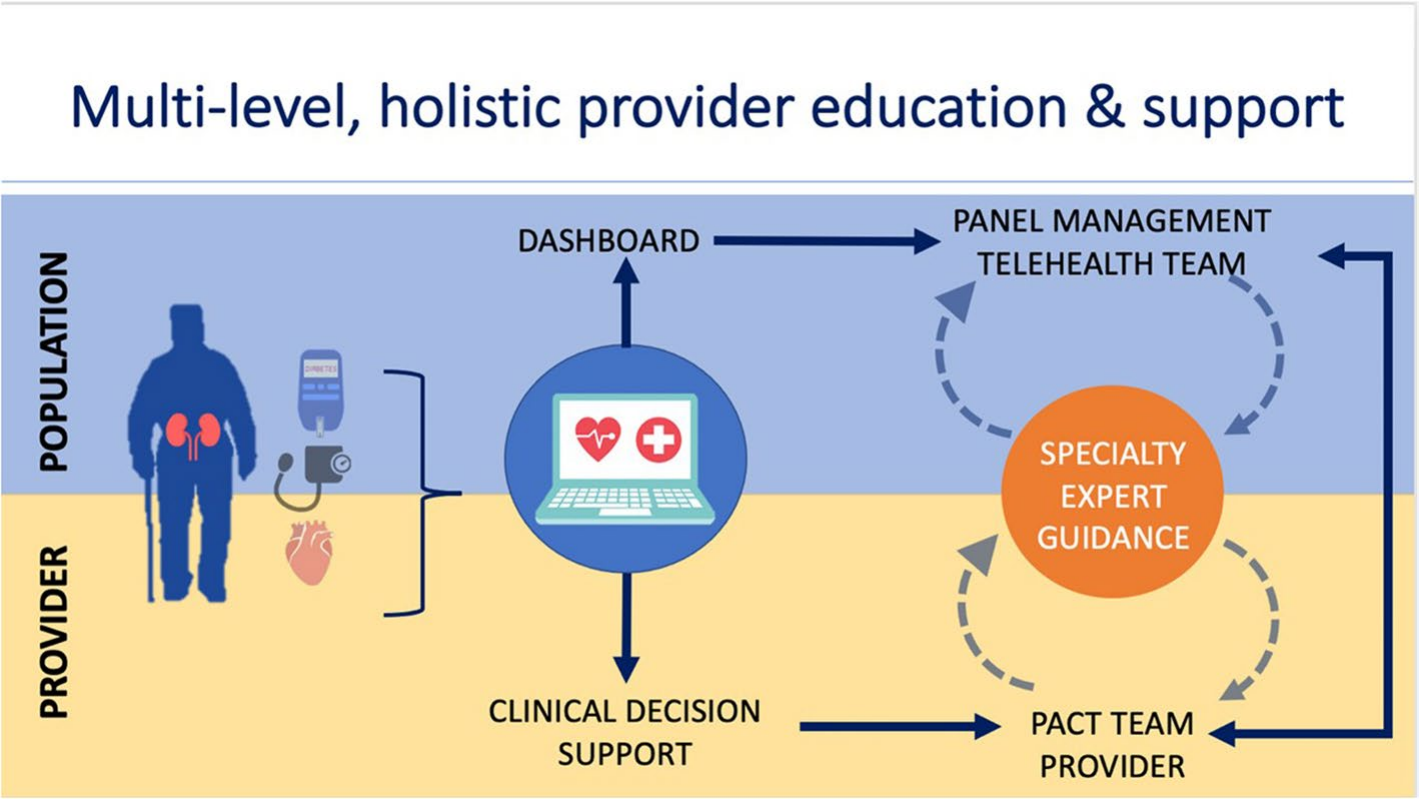
Practice facilitation intervention

A centralized telehealth team comprising a PCP, two telehealth nurses and a clinical pharmacist will facilitate PACT team detection for CKD at the San Francisco VA (Fig. 2). This intervention will leverage health informatic tools and support PCPs’ practice through dashboards, panel management, and a clinical decision support (CDS) system [22, 23]. Specifically, a centralized telehealth team will use a dashboard to screen for patients with hypertension and diabetes at risk of CKD. They will subsequently review management recommendations presented by a CDS tool. The physician leader of the centralized telehealth team (PJ) will prepare recommendations specific to the clinical case. PCP may elect to either receive the recommendations or consideration or to have the centralized telehealth team implement the recommendations, if in accord with patient preferences. These recommendations and any subsequent actions will be documented in the electronic health record (EHR). Patient outcomes will be monitored. The deployment of this intervention exclusively in the San Francisco VA will allow us to assess if the practice facilitation leads to improved testing for CKD above and beyond the proposed educational intervention and the deployment of primary care clinical champions. A key feature of the dashboard and the CDS is that they keep physical and logical independence from the VA electronic health record (CPRS), thus, they can be used with the Cerner electronic health record environment when nationwide VA transition occurs.

##### CKD dashboard and CDS accessible to EHR via service-oriented architecture

The San Francisco VA is part of the VA Sierra Pacific Network, also known as Veterans Integrated Service Network (VISN) 21. The VISN-21 dashboard is an electronic platform developed at the VA Palo Alto HCS which provides physicians with quality metrics for hypertension, diabetes, tobacco use, cardiovascular disease, vaccination, cancer screening, and mental health. Because quality metrics are not readily available for adequately identifying patients with or at risk of CKD, the screening definitions are being redesigned and validated to identify patients more equitably and inclusively. A CKD quality metric filter is being added for patients with hypertension and diabetes to indicate whether triple marker screening and appropriate goal-directed medical management has been achieved. A CDS that was designed by the Medication Safety (MedSafe) QUERI CDS team [24] is being modified in collaboration with C^3^ study team members to include recommendations for triple marker screening and appropriate medication targets. Both the telehealth team and PACT providers at the San Francisco VA Medical Center will have access to the dashboard and CDS. The telehealth team will use the CKD dashboard to identify patients with hypertension and diabetes not meeting guideline-directed medical therapy targets. The telehealth team will synthesize these quality metrics and CDS management recommendations into reports for PCPs and will work with PACTs to facilitate CKD testing and provide recommendations on initiation of cardio-kidney protective therapies. Although initially, the quality metric filter will only be added for patients with hypertension and diabetes, an additional quality metric filter for CKD testing in patients with established CVD will be added during the implementation period.

##### Specific activities

Implementation facilitation strategies have identified common key components of success: goal setting, practice consensus building, and audit and feedback [25]. The telehealth team will reach out to PACT teams to provide overviews of prescribing practices and patient outcomes privately. During meetings, the telehealth team will review treatment goals and offer shared management of patients. Subsequently, the telehealth team will screen patients without optimal management of diabetes and hypertension. Patients not meeting CKD diagnostic or therapeutic targets will be reviewed with the PACT pharmacist and home telehealth team weekly. During weekly meetings, progress will be discussed and recommendations regarding CKD testing will be made.

##### Interdisciplinary expert panel meetings

Recommendations from the telehealth to the PACT teams will involve testing, treatment, and referral recommendations for CKD. As such, an interdisciplinary expert panel of specialty providers composed of two endocrinologists, one nephrologist, one cardiologist, and one pharmacist was formed to meet monthly with the telehealth team. The expert panel provided input on a testing and treatment algorithm for goal-directed therapy to optimize primary and secondary prevention of CKD and cardiovascular disease among patients with diabetes and hypertension. Meetings will review PACT team recommendations to ensure adherence and fidelity to guideline-concordant care.

#### Assessment of intervention effectiveness

The KHRC data registry will be queried to identify patients with T2D, hypertension or cardiovascular disease who had at least one primary care visit in the year prior to the intervention. A pre-post observational design will be used to assess the association of the comprehensive educational intervention with the change in optimal CKD screening separately at each site comparing the proportion of patients tested in the year prior to implementation to the implementation year. In addition, a difference-in-differences analysis will compare the proportion of patients with optimal CKD screening between the San Francisco and San Diego VAs when the intervention combines comprehensive education and practice facilitation. This design uses longitudinal information in the period before and after an intervention and compares with parallel changes in a “control group” in the same time period. Because the practice facilitation strategy will only be implemented at the San Francisco VA, the San Diego VA will serve as the control facility in these analyses. This site was selected due to similar facility VA complexity ratings, comparable patient demographics and geographic proximity, and comparable rates in CKD testing.

The primary level of analysis will be the patient encounter level: proportion of primary care clinic visits for patients with T2D, hypertension and cardiovascular disease resulting in CKD screening.

#### Primary outcome

##### Change in optimal CKD screening

Implementation effectiveness will be assessed by the change in the rate of CKD screening separately at each site for patients with T2D, non-diabetic patients with hypertension, and for non-diabetic patients with established cardiovascular disease. Because cystatin C was only uniformly available in the San Francisco VA prior to C^3^ implementation, optimal CKD screening in the period prior to implementation will be defined as patients with both creatinine-based eGFR and ACR in the San Diego and Houston VAs, and with creatinine eGFR, cystatin C-based eGFR and ACR at the San Francisco VA. In the implementation period, optimal screening for CKD will be defined as patients with the triple-marker screen across the three sites.

##### Secondary outcomes

We will assess the change in the rate of prescription of key cardio-kidney preventive therapies that have been shown to lower the risk of cardiovascular disease and of CKD progression. Specifically, we will assess changes in prescription of angiotensin/converting enzyme inhibitors and angiotensin receptor blockers (ACEI/ARBs), sodium glucose co-transporter 2 inhibitors (SGLT2i) and glucagon-like peptide-1 receptor agonists (GLP1-RA). In addition, as we anticipate that the larger detection of CKD will lead to higher number of nephrology referrals of patients deemed to be at high-risk for CKD progression, we will examine the rate of change in nephrology specialty referrals at each site.

##### Analysis

Multivariable mixed-effects logistic regression will be used to assess the association between the intervention and the change in the rate of optimal CKD screening at each site. Multivariable models will adjust for demographic characteristics, race/ethnicity, co-morbidities and medications. For the difference-in-difference analyses, multiple group propensity score weighting will be used to balance differences across the four groups (pre- and post-time periods at each of the two facilities) on key variables included in the multivariable models, following published methods [26]. Specifically, multivariable models will adjust for the following variables: age, sex, service connected disability or service connection for diabetes, rurality, median income in ZIP Code, ZIP Code social deprivation index, smoking status, alcohol use disorder based on the AUDIT classification, hemoglobin A1C concentration, prescription of other medications for diabetes, body-mass index, diagnosis of hypertension, mental health diagnosis, atherosclerotic cardiovascular disease diagnosis, heart failure diagnosis, COVID-19 diagnosis, and CKD diagnosis.

## Discussion

The enormous current public health burden of CKD is expected to worsen, with CKD projected to become the 5^th^ leading global cause of death by 2040 [27]. Health systems must respond to this global public health challenge by implementing innovative strategies to bridge the enormous current detection and treatment gaps in CKD care. The overarching goal of C^3^ is to improve CKD detection and treatment in the VA healthcare system—one of the largest integrated health systems in the U.S. The first phase of C^3^ described in this protocol aims to achieve universal testing for CKD in high-risk patients across three large academic medical centers responsible for the care of a large racially and ethnically diverse patient population.

The focus of C^3^ is to improve CKD early detection in primary care. In real-world practice, PCPs are at the frontlines of CKD detection and treatment. Yet, despite increasingly complex care required by patients with numerous comorbidities, the median primary care visit is only 15.7 min long [28]. In C^3^, we aim to improve the CKD primary care detection gap by implementing two interventions that are responsive to the known major barriers precluding adequate primary-care-based CKD care: (1) a comprehensive educational intervention responsive to the known knowledge and self-efficacy barriers for CKD testing in primary care; and (2) a practice facilitation intervention that leverages health care informatic tools, and that is responsive to reported time and physical constraints for CKD detection and treatment in primary care. Because the combined educational and facilitation intervention will only be implemented in the San Francisco VA, we will be able to assess the added value of the facilitation intervention against the educational intervention in the other two facilities.

To our knowledge, this is the first study conducted in the U.S which aims to introduce cystatin C as part of the routine detection and staging evaluation of patients with CKD in primary care. For patients with a creatinine-based eGFR less than 60 ml/min/1.73 m^2^, reclassification to a higher or lower GFR category with a cystatin C equation accurately distinguishes persons at higher and lower cardiovascular and CKD progression risk, respectively [14]. Thus, we expect that the routine introduction of cystatin C for CKD detection will result in enhanced risk stratification in these patients [28, 29]. In addition, a recent National Kidney Foundation/American Society of Nephrology task force recommended the removal of the race coefficient from all creatinine-based GFR estimating equations and the enactment of national efforts to facilitate the routine use of cystatin C in the estimation of GFR [30]. These recommendations are consistent with recent evidence that shows cystatin C provides an accurate race-neutral method for GFR estimation [31, 32]. The formative evaluation leveraging qualitative methods will allow us to gain key insights into the acceptability and feasibility of routine cystatin C testing.

Results from the first phase of C^3^ will inform a larger hybrid implementation-effectiveness trial aimed at improving CKD detection and treatment nationwide. If not coupled with action, CKD detection and staging will not be sufficient to achieve markedly higher utilization rates of cardio-kidney recommended preventive therapies and to improve referral of patients that would derive the most benefit from nephrology specialty care. Thus, this C^3^ phase could set the stage for further improvements in CKD detection and treatment in the VA system and, hopefully, across health care systems worldwide.

## Data Availability

The datasets used and/or analyzed during the current study are not publicly available due to internal VA policy. All repositories must remain under control of the VA and must be physically located within space owned or leased by the VA. These data will be released to interested parties for research purposes contingent upon approval by the VA Research and Development office and pursuant to a data use agreement and a memorandum of understanding.

## Abbreviations

C^3^: Chronic kidney disease Cascade of Care
CKD: Chronic kidney disease
eGFR: Estimated glomerular filtration rate
ACR: Urinary albumin to creatinine ratio
ESKD: End stage kidney disease
PCP: Primary care provider
T2D: Type 2 diabetes
VA: Veteran Affairs Healthcare System
KDIGO: Kidney Disease Improving Global Outcomes
PACT: Patient Aligned Care Team
KHRC: Kidney Health Research Collaborative
CDS: Clinical Decision Support
HER: Electronic Health Record
VISN: Veteran Integrated Service Network
ACEI/ARBs: Angiotensin/converting enzyme inhibitors and angiotensin receptor blockers
SGLT2i: Sodium glucose co-transporter 2 inhibitors
GLP1-RA: Glucagon-like peptide-1 receptor agonists
